# Characteristics of cervicovaginal microflora at different cervical maturity during late pregnancy: A nested case-control study

**DOI:** 10.1371/journal.pone.0300510

**Published:** 2024-03-20

**Authors:** Ping Chen, Tingting Hu, Zheng Zheng, Robert E. Garfield, Jinying Yang

**Affiliations:** 1 Department of Gastroenterology, The Second Affiliated Hospital, Guangzhou Medical University, Guangzhou, China; 2 The Guangdong-Hong Kong-Macau Joint Laboratory for Cell Fate Regulation and Diseases, GMU-GIBH Joint School of Life Sciences, Guangzhou Medical University, Guangzhou, Guangdong, China; 3 Guangzhou Women and Children’s Medical Center, Guangzhou, Guangdong, China; 4 Department of Obstetrics and Gynecology, University of Arizona College of Medicine Phoenix, Phoenix, AZ, United States of America; 5 Department of Obstetrics, Longgang District Maternity & Child Healthcare Hospital of Shenzhen City (Longgang Maternity and Child Institute of Shantou University Medical College), Shenzhen, Guangdong, China; Universidad San Francisco de Quito, ECUADOR

## Abstract

**Objective:**

The mechanism of cervical ripening in late pregnancy is still unclear. The vaginal microbiome has been reported to correlate with the preterm birth and short cervix in pregnant women. However, the associations between the cervical maturity and the vaginal microbiome are still poorly understood. We aim to analyze the cervicovaginal microflora in women with ripe cervix and in those who are unripe when delivering at term.

**Methods:**

Cervicovaginal swabs were collected between 40 and 41 weeks of gestation from the following 2 different groups of patients: ripe group (n = 25) and unripe group (n = 25). Samples were tested using 16S ribosomal RNA gene high-throughput sequencing and analyzed by bioinformatics platform.

**Results:**

This study highlights the relationship between cervical maturity during late pregnancy and the composition of the cervicovaginal microflora. Both α- and β-diversity analyses demonstrated significant differences between women with a ripe cervix and those with an unripe cervix. Notably, the *Lactobacillus* profile was found to be closely linked to cervical maturity. There was a significant difference in the vaginal community state type, with CST IV being more prevalent in women with an unripe cervix. Furthermore, the association between CST IV and the unripe cervix group, as indicated by the odds ratio of 8.6, underscores its relevance in evaluating cervical maturity, when compared to other *Lactobacillus*-dominant community state types. Additionally, several bacterial taxa, particularly *Lactobacillus*, exhibited differential relative abundances between the two groups.

**Conclusion:**

This study provided significant evidence regarding the relationship between the vaginal microbiome and cervical maturity, highlighting the differential diversity, community state types, and specific bacterial taxa, such as *Lactobacillus*, that are associated with cervical maturation status. These findings contributed to our understanding of the dynamics of the cervicovaginal microflora during late pregnancy and its implications for cervical health.

## Introduction

Cervical ripening is a crucial physiological process that occurs before childbirth, involving the softening and decreased tension of the cervix, gradual shortening until disappearing, and dilation of the uterine orifice for smooth delivery. The maturity of the cervix plays a vital role in predicting the final delivery method and directly affects pregnancy outcomes, labor progress, and the success of inducing labor. Insufficient cervical maturity can cause a significant increase in the rate of cesarean sections [1]. Moreover, inducing labor at a time of low cervical maturity may result in prolonged labor and compromise fetal well-being. Hence, cervical maturation plays a crucial role in normal and induced labor by promoting cervical ripening and serves as a bottleneck to increasing the rate of normal delivery [2].

The mechanism of cervical ripening is complex and not yet fully understood. Studies suggest that cervical maturation is accompanied by changes in hormones, local immune regulation, and inflammatory response processes [3, 4]. With the advancement of high-throughput sequencing technology, evidence increasingly suggests that a small but diverse microbial community exists in the female reproductive system, including the vagina, cervix, and uterine cavity [5, 6]. The interaction between these microbial communities and their host has been mechanistically linked to health and disease pathogenesis [7–11]. Recently, some studies have focused on the role of the cervicovaginal microbiota in pregnancy outcomes, examining the relationship between these microbial communities and preterm birth [5, 12–14] or short cervix [15]. Microorganisms participate in cervical ripening through two ways: bacterial endotoxin LPS stimulates the cytokine network system, which promotes TNF, IL-1, IL-6, and IL-8 secretion, and IL-8 is considered a primary factor closely related to cervical ripening. It can activate collagenase, elastase, degrading enzymes, metalloproteinases, hyaluronidase, and other enzymes that promote cervical ripening. Bacteria can also produce exotoxins like hyaluronidase, collagenase, streptokinase, and other enzymes closely linked to cervical ripening [16–18].

The study of the microbiome related to cervical ripening is significant because it can help determine if the cervicovaginal microbiota is directly linked to cervical ripening or through microbial metabolites. However, the relationship between microorganisms in the reproductive system and cervical ripening in full-term pregnancies remains unknown. In this study, we collected cervicovaginal samples and established an appropriate cohort to characterize the cervicovaginal microbiota and assessed differences in the cervicovaginal microbiota composition between women with ripe and unripe cervix. These data offered a new avenue for studying mechanisms of cervical ripening by associating cervicovaginal microbiota with cervical maturity and provided potential opportunities to modify cervical maturity through regulation of the cervicovaginal milieu.

## Materials and methods

### Study population and sample collection

This nested case-control study was performed with 500 singleton pregnancy women who registered in Guangzhou Women and Children’s Medical Center, for a routine prenatal test from 1 December 2020 to 31 December 2021. Fifty biospecimens were obtained between 31 December 2020 and 1 December 2021 for the analysis in our study. The study was approved by the Institutional Review Board of the Guangzhou Women and Children’s Medical Center (#202057401). Participants were recruited from women of the Han nationality who had been hospitalized at Guangzhou Women and Children’s Medical Center. Low-risk nulliparous women who were at 40 weeks 0 days to 40 weeks 6 days of gestation (calculated from the date of the first day of the last menstrual period and confirmed by ultrasound scan) with a live singleton fetus in cephalic presentation, who had no contraindication to vaginal delivery, and who had no cesarean delivery planned were included in this study. Low risk was defined as the absence of any condition considered to be a maternal or fetal indication for delivery before 40 weeks 5 days (e.g., hypertensive disorders of pregnancy or suspected fetal-growth restriction). Participants would be excluded if they were in labor or had premature rupture of membranes or vaginal bleeding or non-reassuring fetal status requiring immediate delivery.

Furthermore, women with activity in the vagina in the 24 hours prior to specimen collection (including sexual intercourse, a transvaginal ultrasound, digital cervical exam, or vaginal lubricants) were excluded. Finally, for the present discovery study, cases (ripe group) were defined as women with cervix’s Bishop Score between ≥6 (n = 25), and controls (unripe group) were defined as women with cervix’s Bishop Score 0 to 4 (n = 25). All the cervicovaginal specimens were collected under direct vision by the same attending obstetrician using a sterile Dacron swab from the posterior vaginal fornix before the vaginal digital examination (Bishop scoring). Samples were collected in 0.5 mL phosphate-buffered saline, immediately placed in liquid nitrogen, and stored at -80°C for future use.

### DNA extraction and 16S rRNA gene sequencing

Total genome DNA from samples was extracted using CTAB method. DNA concentration and purity were monitored on 1% agarose gel [19]. According to the concentration, DNA was diluted to 1 ng/μL using sterile water. The V3-V4 regions of 16S rRNA genes were amplified using 341F (5’-CCTACGGGRBGCASCAG -3’) and 806R (5’-GGACTACNNGGGTATCTAAT -3’) primer with the barcode. All PCR reactions were carried out in 30 μL reactions with 15 μL of Phusion® High-Fidelity PCR Master Mix (New England Biolabs); 0.2 μM of forward and reverse primers, and about 10 ng template DNA. Thermal cycling consisted of initial denaturation at 98°C for 1 min, followed by 30 cycles of denaturation at 98°C for 10 s, annealing at 50°C for 30 s, elongation at 72°C for 60 s, and finally 72°C for 5 min. The amplified PCR products of between 400 to 450 bp in size from each sample were pooled in equimolar concentrations. Then, mixture PCR products were purified with AxyPrepDNA Gel Extraction Kit (AXYGEN). The 16S rRNA gene sequencing was performed by Shanghai Applied Protein Technology Co. Ltd. Sequencing libraries were generated using NEB Next®Ultra™DNA Library Prep Kit for Illumina (NEB, USA) following manufacturer’s recommendations and index codes were added. The library quality was assessed on the Qubit@ 2.0 Fluorometer (Thermo Scientific) and Agilent Bioanalyzer 2100 system. At last, the library was sequenced on a novaseq 6000 PE250 platform and 250bp paired-end reads were generated.

### Bioinformatic analysis

Sequenced data were demultiplexed according to the unique barcodes then quality-controlled using FastQC [20]. Forward and reverse end sequences of respective samples were merged using FLASH [21]. Sequences analysis was performed by QIIME (Quantitative Insights Into Microbial Ecology, version 1.80) pipeline [22]. Operational taxonomic units (OTUs) were generated by UPARSE-OTU and UPARSE-OTU ref algorithms with ≥97% similarity. We picked representative sequences for each OTU and use the RDP classifier with SILVA 132 reference database to annotate taxonomic information for each representative sequence. To correct the sampling error, Subsamples of minimal number of sequences (51,174 sequences) per sample were randomly selected to compare the alpha and beta diversity between samples. Shannon diversity estimators, Chao1 richness estimators, Simpson’s diversity index, PD whole tree, and goods’ coverage were measured by R software [23] with the vegan package [24]. The differences in the beta diversity were presented as principal coordinate analysis using the QIIME pipeline. The principal coordinate analysis (PCoA) method and Mantel test were performed in QIIME. To confirm differences in the abundance of individual taxonomy between the two groups, STAMP software was utilized [25]. LEfSe was used for the quantitative analysis of biomarkers within different groups [26]. This method was designed to analyze data in which the number of species is much higher than the number of samples and to provide biological class explanations to establish statistical significance, biological consistency, and effect-size estimation of predicted biomarkers. To identify differences in microbial communities between the two groups, Wilcoxon test, Anosim was performed based on the weighted UniFrac distance matrices. The significant statistical test, hierarchical clustering, and heatmap analysis were performed by R. The community state types (CSTs) were performed as previously described [27, 28] by using Hierarchical clustering analysis of vaginal microbiota profiles with Jensen-Shannon metric of Ward linkage. We tabulated the proportion of women whose pregnancy vaginal sample was categorized into each CST and compared the proportion of women with ripe group and the proportion of women with unripe group across the CST categories using the Chi-square test. To evaluate the association between the CST types in cervical maturity, the logistic regression was also performed, contrasting ripe group with unripe group, according to vaginal CST category.

## Results

### Description of participant

Cervicovaginal swab samples were collected from a total of 50 women, including 25 women with unripe cervixes (Bishop Score between 0 to 4) and 25 age-matched women with ripe cervixes (Bishop Score ≥6). The clinical and demographic characteristics of the women included in this study were presented in Table 1. At the time of enrollment, there were no significant differences in age, height, weight, body mass index, gestational age, nulliparity, or neonatal birth weight between the ripe and unripe groups, with all P>0.05 (Table 1). All participants were of Chinese Han ethnicity, with no difference in race.

**Table 1:** Summary of baseline demographics.

	Ripe group	Unripe group	P
Age at conception in years; Median (IQR)	30 (26–32.75)	29 (26–31.75)	0.696
Outcome evaluation of gestational Age(days); Median (IQR)	286 (285–286)	286 (285–286)	0.637
Height at conception in cm mean (IQR)	160.97 (157.00–165.00)	161.23 (157.75–165.00)	0.539
Weight at conception in kilograms mean (IQR)	68.56 (63.00–73.75)	68.84 (63.75–75.00)	0.894
BMI at conception; Median (IQR)	26.38 (25.31–27.84)	26.04 (24.81–27.73)	0.697
Birth weight in grams mean (IQR)	3422.9 (3157.50–3655.00)	3362.9 (3100.00–3637.50	0.554

Abbreviations: IQR, interquartile range.

### 16S RNA gene sequencing

We characterized the vaginal microbiota using pyrosequencing of barcoded 16S RNA genes. The data set consisted of 4,347,630 high-quality paired-end reads after the quality control steps, with the median number of sequences per sample was 79,134 (IQR: 67,394 to 98,663) and the good coverage of samples ≥99.89%. A rarefaction curve displaying rarefaction depth by Shannon diversity was given in Supplement **S1 Fig**. After removing singleton OTUs, these sequences could be divided into 774 OTUs at a 0.03 distance, and median number of OTU in each sample was 95 (IQR: 76 to 128) OTUs. Four OTUs were distributed in all samples accounting for 75.11% of total reads. They belonged to *Lactobacillus crispatus*, *Lactobacillus iners*, *Gardnerella vaginalis*, and unclassified *Aquabacterium* with the average relative abundance of 39.33, 26.91, 7.73, and 1.15%.

### Vaginal community state types associated with cervical ripening

**Fig 1:**
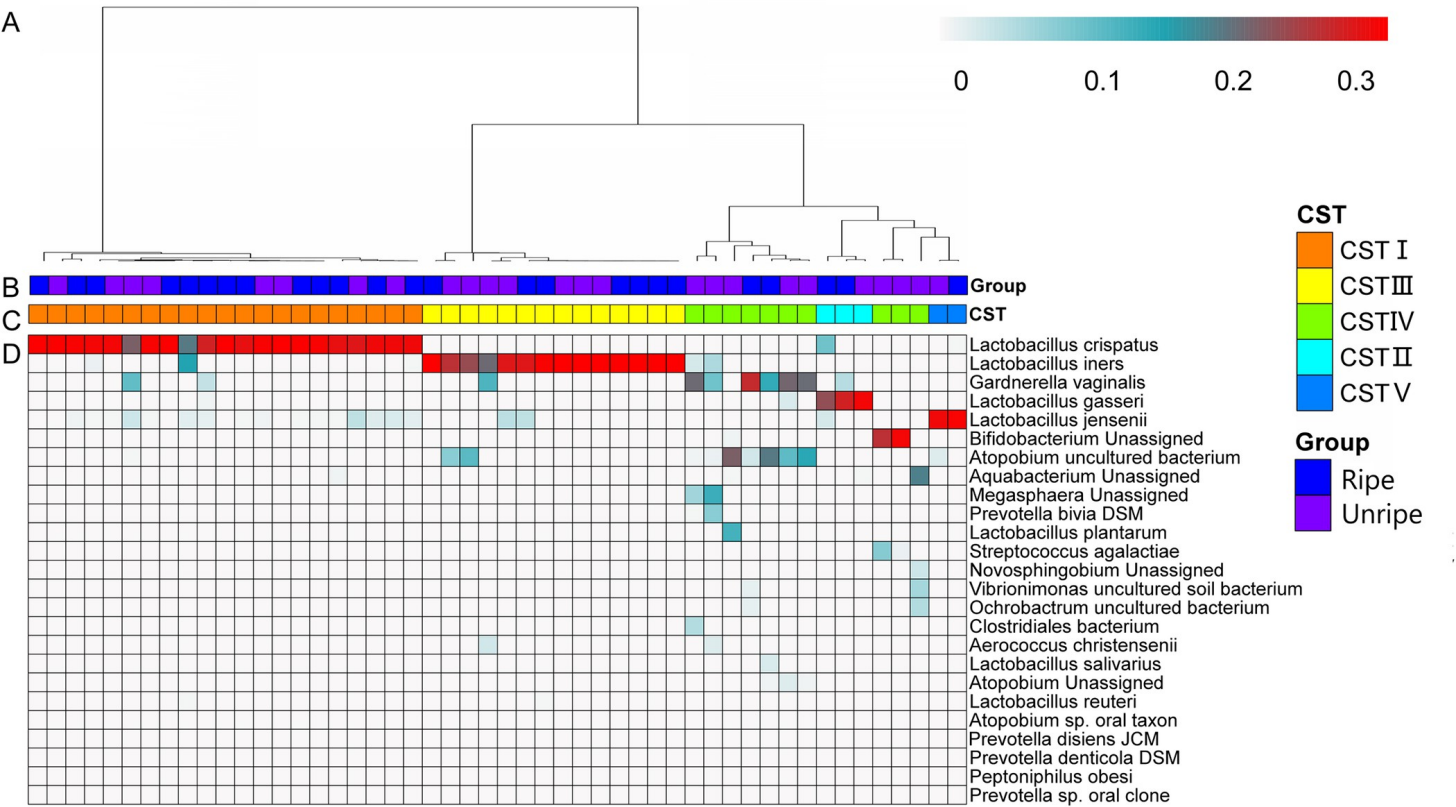
Heatmap of microbial taxa relative abundance identified in the vaginal microbiota comunities of ripe and unripe group. (A) Hierarchical clustering of jensen-shannon distance matrices with Ward linkage on relative abundances of reads for each OTU within individual samples. (B) Mature group shown in bule, unripe group shown in purple. (C) Community state types (CST) identified across all the study subjects. Each CST was represented by a different color according to the key shown underneath. (D) Heatmap of relative abundances of bacterial species within the vaginal microbiota of each woman with log10 transformation. Each column represented a woman’s vaginal microbiota profile, and each row represented a bacterial species. Only represent that relative abundance of the 25 most abundant species in the vaginal microbiota that define community state types were shown above the heatmap.

Hierarchical clustering analysis of vaginal microbiota profiles using Jensen-Shannon metric with Ward linkage (Fig 1) were conducted in community state types (CSTs) as previously described [27, 28]. A heatmap representing the composition of the vaginal microbiome, as classified by CST, according to the different state of cervix is given in Fig 1. Community states clustered into mainly three groups with similar bacterial composition and abundance. The most commonly observed was CST I (*Lactobacillus crispatus*-dominated) followed by CST III (*Lactobacillus iners*-dominated), CST IV (a lower proportion of lactic acid-producing bacteria and higher proportion of anaerobes such as the genera *Atopobium*, *Gardnerella*, *Peptoniphilus*, *Prevotella*), CST II (*Lactobacillus gasseri*-dominated) and CST V (*Lactobacillus jensenii*-dominated). Comparing the CST types of ripe and unripe groups, CST IV was significantly overrepresented in the unripe group compared to ripe group (P = 0.034, Chi-Square test). Frequencies of observed CST across cervical ripening and immaturity participants were described in detail in Table 2. The logistic regression analysis also confirmed that those in the CST IV had substantially and significantly elevated odds of unripe cervix (OR = 8.6 [1.3, 55.6]).

**Table 2:** Frequencies of bacterial CSTs between ripe group and unripe group.

Vagitype	No. with characteristic/no. total (%)			
	Overall (frequency)	Ripe group	Unripe group	P value^a^
CST I (*L*. *crispatus*)	21/50 (42%)	13/25 (52%)	8/25 (32%)	0.152
CST II (*L*. *gasseri*)	3/50 (6%)	2/25 (8%)	1/25 (4%)	0.552
CST III (*L*. *iners*)	14/50 (28%)	7/25 (28%)	7/25 (28%)	1.000
CST V (*L*. *jensenii*)	2/50 (4%)	1/25 (4%)	1/25 (4%)	1.000
CST IV	10/50 (20%)	2/25 (8%)	8/25 (32%)	0.034
*Lactobacillus* profile	40/50 (80%)	23/25 (92%)	17/25 (68%)	

### The cervicovaginal microbiota of women who with cervical ripening versus those who with cervical immaturity

**Fig 2:**
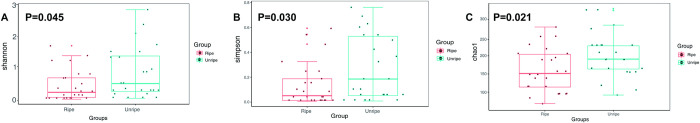
Species diversity between study groups. Alpha diversity comparison as measured by Shannon (A), Simpson (B) and Chao1 index (C). Significant differences as calculated by Wilcoxon’s test (P<0.05).

**Fig 3:**
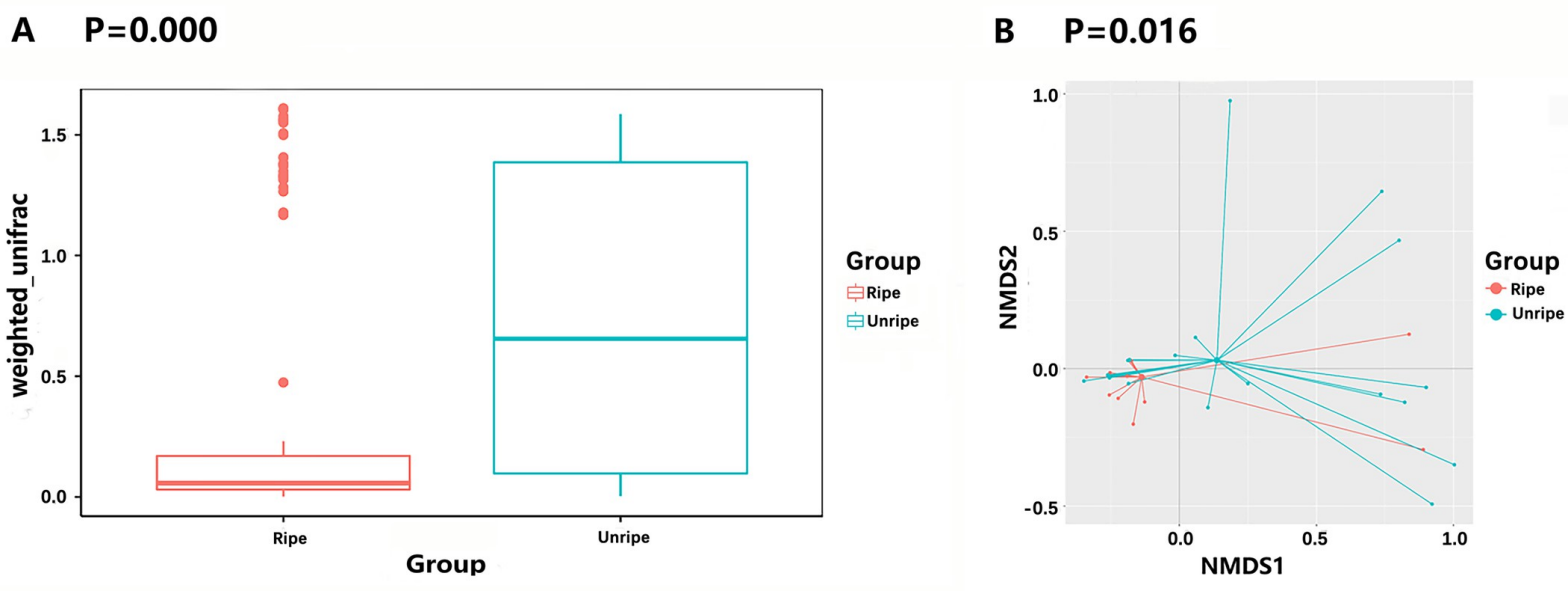
Beta diversity between vaginal microbiomes of the groups was measured by Weighted Unifrac Distance (A) and visualized using non-metric multidimensional scaling (NMDS) (B). Significance of group dissimilarity as calculated by Wilcoxon’s test and Anosim were identified by the given P-value.

The microbiome structures of participants who revealed cervical maturity were compared by investigating their α- and β-diversity at the 0.03 OTU level. There was a significant difference in α- diversity measure between the ripe or unripe groups using Shannon, Simpson, and Chao1 measures (P<0.05, Fig 2A–2C). The β-diversity measured by weighted unifrac distance was also significantly different between these two groups when calculated either by Anosim or Wilcoxon test (P = 0.000, Fig 3). Non-metric multidimensional scaling (NMDS) analysis shows clear separation among the microbial communities from the cervical maturity participants and the cervical immaturity participants (Fig 3B). The stress score of the resulting NMDS was quite low (0.060), indicating the information loss was low.

**Fig 4:**
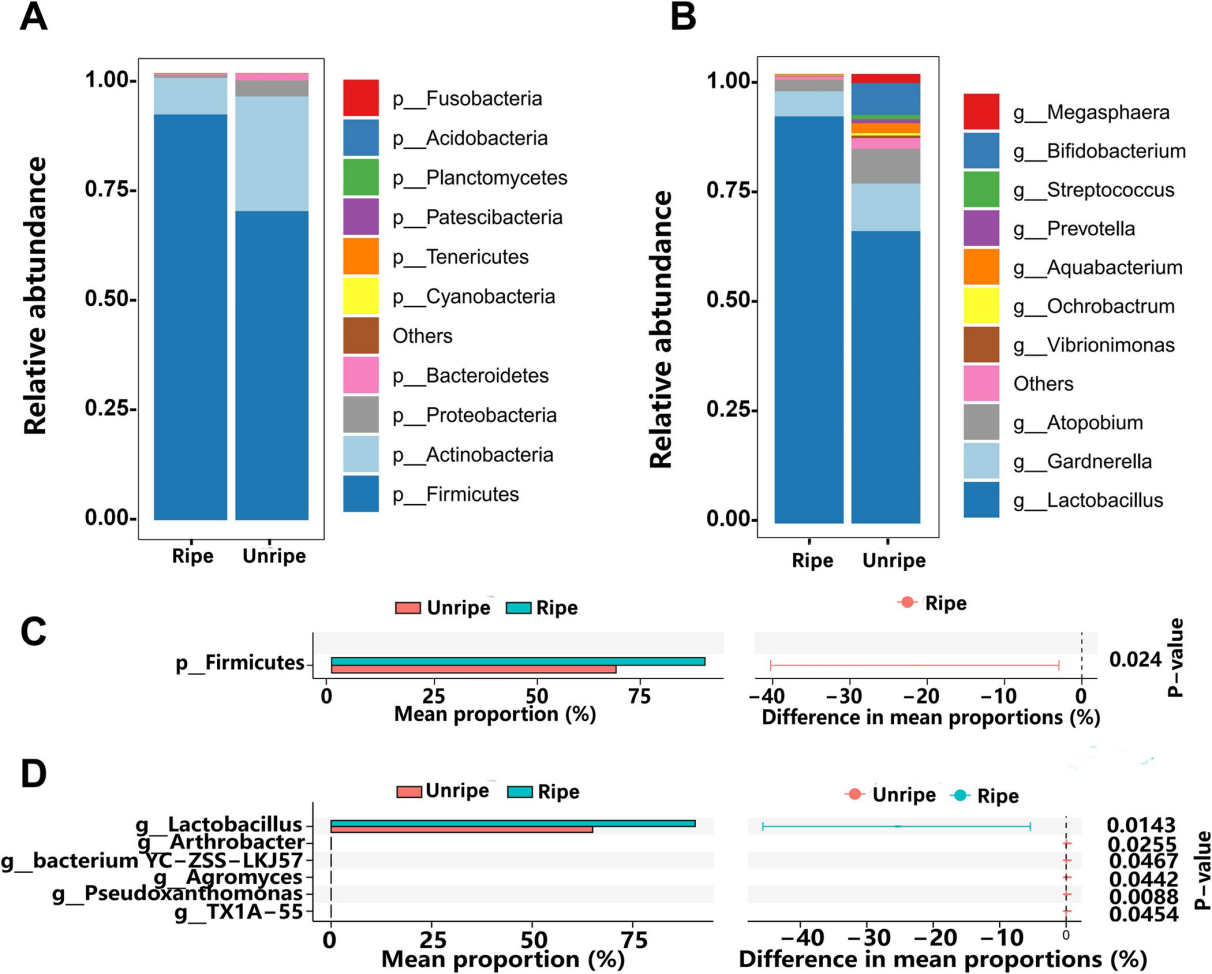
Vaginal microbiome composition in women across study groups. The relative abundance of detected phyla (A) and genera (B) in groups of women who had ripe cervix and unripe cervix. Comparitive analysis between the groups for the phyla(C) and genera (D) across all samples. Significant differences were calculated using Welch’s t-test by STAMP.

Given the high diversity of microbes in samples and the significant difference between ripe and unripe groups, we next analyzed whether any microbial taxa were particularly associated with cervical maturity. We first compared the relative abundance at the phyla level between ripe and unripe groups (Fig 4A and 4C). Taxonomic classification revealed that a total of 24 phyla were observed from the samples. Among them, *Firmicutes*, *Actinobacteria*, *Proteobacteria*, and *Bacteroidetes* were distributed in all samples. *Firmicutes* were the most abundant in both groups at 79.93% mean relative abundance, with a greater proportion observed in ripe group (90.73%) compared to unripe group (69.13%) (Fig 4A). And this difference was significantly based on the significant statistical test (Fig 4C). *Actinobacteria*, *Proteobacteria*, and *Bacteroidetes* represented 16.92, 2.12, and 0.90% mean relative abundance, with a greater proportion observed in unripe group (25.64, 3.58, 1.53%), compared to ripe group (8.19, 0.66, 0.27%) (Fig 4A).

At the rank of class, there were six classes, *Bacilli*, *Actinobacteria*, *Gammaproteobacteria*, *Bacteroidia*, *Alphaproteobacteria*, and *Clostridia* distributed in all samples. And *Bacilli*, *Actinobacteria*, *Coriobacteriia*, *Gammaproteobacteria*, and *Negativicutes* were relatively abundant in the bacterial community (>1% of the total reads), with 78.49, 11.70, 5.21, 1.40 and 1.06% (Ripe: 90.56, 5.63, 2.56, 0.36, 0.03% vs Unripe: 66.43, 17.77, 7.86, 2.44, 2.10%) mean relative abundance. However, only *Bacilli* was significantly different from ripe group and unripe group among these classes. Meanwhile, two rare classes (0.0001, 0.0001%) *Holophagae*, and *Rhodothermia* were also significantly enriched in unripe group and respectively distributed in 5 and 4 samples of unripe groups (S2A Fig).

At the order level, *Bacteroidales*, *Betaproteobacteriales*, *Bifidobacteriales*, *Clostridiales*, *Lactobacillales*, and *Sphingomonadales* were distributed in all samples. And *Lactobacillales*, *Bifidobacteriales*, *Coriobacteriales*, *Betaproteobacteriales*, and *Selenomonadales* were relatively abundant in the bacterial community (>1% of the total reads), with 78.48, 11.65, 5.21, 1.22 and 1.07% (Ripe: 90.56, 5.60, 2.56, 0.15, 0.03% vs Unripe: 66.41, 17.70, 7.86, 2.29, 2.10%) mean relative abundance. However, only *Lactobacillales* was significantly different from ripe group and unripe group among these orders. At the same time, four rare orders (0.0003, 0.0001, 0.0001, and 0.0001%) of PLTA13, *Balneolales*, *Acidithiobacillales* and *Holophagae* subgroup 7 were also significantly different between ripe group and unripe group (S2B Fig).

At the rank of family, *Bifidobacteriaceae*, *Burkholderiaceae*, *Lachnospiraceae*, *Lactobacillaceae*, *Ruminococcaceae*, and *Sphingomonadaceae* were distributed in all samples. And *Lactobacillaceae*, *Bifidobacteriaceae*, *Atopobiaceae*, *Burkholderiaceae*, and *Veillonellaceae* were relatively abundant in the bacterial community (>1% of the total reads), with 77.78, 11.65, 5.17, 1.22, and 1.06% (Ripe: 90.54, 5.60, 2.56, 0.15, 0.03% vs Unripe: 65.03, 17.70, 7.78, 2.29, 2.10%) mean relative abundance. However, only *Lactobacillaceae* was significantly different among ripe group and unripe group among these families. At the same time, five rare families (0.0123, 0.0019, 0.0001, 0.0001, and 0.0001%) of *Micrococcaceae*, *Bacterium* YC-ZSS-LKJ57, *Balneolaceae*, *Peptococcaceae*, and *Acidithiobacillaceae* were also significantly different between ripe group and unripe group (S2C Fig).

At the rank of genus, *Lactobacillus*, *Gardnerella*, and *Aquabacterium* were distributed in all samples. And *Lactobacillus*, *Gardnerella*, *Atopobium*, *Bifidobacterium*, *Aquabacterium* and *Megasphaera* were relatively abundant in the bacterial community (>1% of the total reads), with 77.78, 8.07, 5.17, 3.57, 1.15 and 1.02% (ripe group, 90.54, 5.59, 2.56, 0.01, 0.12, 0.0002% vs unripe group, 65.30, 10.56, 7.78, 7.14, 2.18, 2.03%) mean relative abundance (Fig 4B). However, only *Lactobacillus* was significantly different from ripe group and unripe group among these genera. At the same time, Five other rare genera were also significantly different (Fig 4D).

At the rank of species, *Lactobacillus crispatus* (39.33%), *Lactobacillus iners* (26.94%), and *Gardnerella vaginalis* (8.07%) were distributed in all samples, and *Lactobacillus gasseri* (5.19%) was distributed in 46 samples, which were relatively abundant in the bacterial community (>1% of the total reads). However, no significant differences were observed between the ripe group and unripe group in terms of these species. Three rare species of *Bacterium* YC-ZSS-LKJ57, *Bifidobacterium longum*, and *Desulfovibrio fairfieldensis* were significantly different between ripe group and unripe group, and they were detected in 36, 8, and 6 samples (S2D Fig).

Overall, women who exhibited cervical maturity showed a significantly higher likelihood of having a *Lactobacillus*-dominated profile and correspondingly lower levels of other non-*Lactobacillus* microbiome profiles compared to those with unripe cervix (Fig 1). A taxon analysis of these profiles confirmed a significantly higher abundance of *Lactobacillus* (P = 0.01) (Fig 4) in the cervical maturity group and lower abundance of *Arthrobacter*, *Agromyces*, *Pseudoxanthomonas*, and so on, which were all significantly enriched in the group with unripe cervix (Fig 4).

## Discussion

The study’s principal findings are as follows: 1) It was a first study to analyze the diversity and comprehensive community structure of cervicovaginal bacteria based on the cervical maturity, our study found that significant differences in the α- and β-diversity between women who had ripe cervix and those who had unripe cervix. 2) A significantly high frequency of vaginal CST IV was observed in women whose cervix was unripe. 3) *Lactobacillus* profile dominance was related to the physiological maturity of the cervix during late pregnancy.

The cervicovaginal microbiome resembles those reported by previous vaginal microbiota studies which are physiologically dominated by *Lactobacillales*, while *Clostridiales*, *Bacteroidales*, and *Actinomycetales* are also regularly detected [14]. These orders were distributed in all samples and the *Lactobacillales* were the most dominant taxon in our data (Figs 1 and 4). It was consistent with that several cohort studies have shown that healthy pregnant women with normal outcomes generally might need a vaginal microbiota dominated by *Lactobacillus* spp. throughout the entire pregnancy [14, 29, 30]. Ethnicity and regional differences are generally believed to play an important role in the vaginal microbiota [13]. There is a consensus that whilst Caucasian populations harbor *Lactobacillales crispatus*, Hispanic and African-American populations harbor a blend of microbiomes, as Asian populations predominantly harbor *Lactobacillales iners* [5, 31]. All samples in our study were collected from Chinese Han women, who are of Asian populations. To compare the cervicovaginal microbial composition of women whose ancestries are European and American [5, 32–34], data in this study revealed the cohort of Han women may have a different vaginal microbial composition. It was in line with previous findings that *Lactobacillus iners* (CST III) were common in Asian women [4], but *Lactobacillus crispatus* (CST I) seemed to have higher frequency in Han women (Table 2). Though the causes of the difference are still unclear, the differences in genetics, geographic location, nutrition, and cultures among ethnicities are likely to contribute to different results [4, 5, 31].

CST I, II, III, and V are dominated by one of four *Lactobacillus* species commonly found in the vagina (*Lactobacillales crispatus*, *Lactobacillales gasseri*, *Lactobacillales iner*, and *Lactobacillales jensenii*), while CST IV comprises of an array of strict and facultative anaerobes and a lower proportion of *Lactobacillus* [28]. *Lactobacillales crispatus* (CST I) and *Lactobacillus iners* (CST III) were the most dominant CST types in our cohorts, whose frequencies were also not significantly different in two groups. However, It revealed relatively high frequency of CST I type in maturity participants (52%) more than in immaturity participants (32%), though there was no significant difference between the two groups base on the statistical tests. It was agreed that *Lactobacillus crispatus* was the protective *Lactobacillus* species, which had been shown to be positive for full-term birth [14, 35, 36].

Among the species of *Lactobacillus* spp, *Lactobacillales iners* was distributed in all samples and revealed the same frequency as CST III type between two groups of the data we present here (Table 2). It had been reported as the most prevalent *Lactobacillus* spp. in the vaginal microbiota and has been previously reported as the most dominant *Lactobacillus* spp. detected in Asian women [28]. *Lactobacillales iners*, despite being a common vaginal commensal bacteria, has been reported to be significantly associated with vaginal colonization and a shortened cervix [37]. The species of *Lactobacillus* spp have also been found to relate with preterm birth and have also been linked to "intermediate" vaginal community states (a vaginal microbiota that may indicate a shift toward bacterial vaginosis) and bacterial vaginosis [38–41]. However, it remains a lingering question whether the presence of *Lactobacillus iners* indicates a healthy or imbalanced state of the vaginal microbiota [42]. Other studies have also reported that women who deliver at term tend to have a vaginal environment dominated by *Lactobacillus iners*. Other studies have also reported that women who deliver at term tend to have a vaginal environment dominated by *Lactobacillus iners* [43]. This finding supports the notion that *Lactobacillus iners* may indeed be indicative of a healthy microbiota. The relative abundance and the frequency of *Lactobacillales iners* did not change significantly between the ripe and unripe groups (Table 2). Of note, *Lactobacillales iners* is the only vaginal *Lactobacillus* spp capable of producing a cytolytic enzyme, inerolysin, which it uses to lyse erythrocytes [44]. The prevalence of *Lactobacillales iners* in the present study was in line with the need for cytolytic enzymes for cervical remodeling during delivery.

In addition to CST I, the Diverse (CST IV) type was found to be dominant in women with cervical immaturity. Our data also revealed that this CST type was more strongly associated with the vaginal microbiome in women with cervical immaturity (Table 2). This CST type as a low proportion of *lactobacilli* includes an increased abundance of mixed species, which has been associated with poorer outcomes [45, 46]. The previous study reported that the composition of the vaginal microbiota during normal pregnancy changed as a function of gestational age, with an increase in the relative abundance of four *Lactobacillus* spp., and decrease in anaerobe or strict anaerobe microbial species as pregnancy progressed [47]. The CST IV type has been found to associate with increased risk of urogenital disease, including sexually transmitted infections and pelvic inflammatory disease [12]. And CST IV type has also been suggested to relate to premature cervical shortening, clinically manifested as a short cervix, and ultimately spontaneous preterm birth [15]. CST IV may affect the secretion of related hormones, which can affect the normal cervical remodeling process. It seemed if the change did not regulate well then it would remain in Diverse (CST IV) and revealed as vaginal disease, which would be bad for the pregnancy outcomes including the cervical ripening for modeling to delivery [12].

As previously mentioned, our study revealed that *Lactobacillus* spp, belonging to *Firmicutes*, *Bacilli*, *Lactobacillales*, *and Lactobacillaceae*, were significantly different in the relative abundance of different cervical maturity degree between the two groups, in line with previous findings that associated with reproductive health and pregnancy outcome [12]. Significant differences were shown in relative abundance from phyla to genera levels, which may indicate their important roles in cervical maturity (Fig 4 and S2 Fig). The current data is insufficient to elucidate the precise mechanism by which the cervicovaginal microbiota actively participate in the process of cervical maturity. However, we speculate that *Lactobacillus* may contribute to cervical maturity through various mechanisms. Firstly, the metabolites produced by *Lactobacillus* could aid in collagen degradation, facilitating cervical remodeling. Secondly, *Lactobacillus* helps maintain a normal acidic environment in the vagina, which is essential for the cervical microenvironment. Lastly, *Lactobacillus* is believed to regulate the immune microenvironment of the cervix, further promoting cervical maturity. Based on these preliminary findings, our future research endeavors involve expanding the sample size across multiple centers to validate and corroborate our results. We plan to integrate metabolomic, proteomic, human immunomic, and clinical data to gain a comprehensive understanding. Additionally, we will conduct animal model experiments to further explore the intricate mechanisms underlying the promotion of cervical maturity by the *Lactobacillus* profile.

Among the *Proteobacteria*, there were *Pseudoxanthomonas* and TX1A-55 at genus level revealed significantly different. One actinobacterial genera, *Arthrobacter* was enriched in women with cervical immaturity. *Actinobacteria* was the regularly detected taxon [13] and reported as the possible causative pathogens in the spontaneous preterm birth without premature rupture of membranes [5, 31]. The enrichment of Actinobacterial taxon in women with cervical immaturity may indicate the imbalance of cervicovaginal flora that hinder the cervix ripening and softening. *Bifidobacterium longum* was presented in only unripe group with rare relative abundance. *Bifidobacterium longum* [48], a prevalent probiotic strain, has been linked to conditions such as obesity, diabetes, and allergies at various life stages due to reduced levels. This bacterium demonstrates lactic acid and butyric acid, antibacterial, antiviral, and anti-inflammatory properties, along with the ability to improve glycemic control, lower blood lipid levels, enhance immunity, exhibit antioxidant activity, prevent eczema, and alleviate stress and allergies. It may contribute to regulating the cervical microenvironment and facilitating normal cervical maturation. *Desulfovibrio fairfieldensis* [49] is associated with inflammatory bowel disease and may impact cervical maturation progression through inflammation. Surprisingly, its abundance is lower in the immature cervix group, necessitating further investigation into the underlying reasons. *Bacterium YC-ZSS-LKJ57* is an unclassified bacteria strain, and its specific physiological function remains unclear as it has not been cultured yet. Most of taxa correlated to the cervix maturity were different from the reported phylotypes that related to preterm birth. Of note, except for the dominant *Lactobacillus*, other significant different genera were rare with <0.1% relative abundance, which corresponded with the vaginal microbiota correlated to spontaneous preterm birth [50].

In the present research, we determined the composition of the vaginal microbiota of women who had ripe cervix and compared these profiles to those of women who have unripe cervix. To minimize any batch effects, we were rigorous in implementation of consistent sample processing and did extensive analysis of the clinical and demographic characteristics to ensure they were well matched (Table 1). The cohorts were comparable in terms of maternal age, BMI, ethnicity and gestational Age, which have been previously reported that might be associated with vaginal microbiota [50].

As far as we know, this is the first study to report the relationship between the vaginal microbiome and cervical maturity. Our findings around the new topic are crucial in contextualizing and interpreting vaginal microbiome and birth outcome studies. We will further build a large multicenter cohort to collect the cervicovaginal sample in different gestation to analyze the effects of cervicovaginal microbiota on cervical maturity dynamically. And we will also measure cervicovaginal cytokine cervical PG, hormone levels including progesterone and relaxin to systemically study the factor contributing to cervical maturity, not just cervicovaginal microbiome but combining metabolomic, proteomic, human immunomic, clinical data and animal model experiments. Conclusions

In summary, our study indicated notable differences in the microbiome composition between women with ripe and unripe cervix. These differences manifested through significant variations in α-diversity measures (including Shannon diversity, Simpson index, and Chao1 estimated species richness), β-diversity patterns, as well as a higher prevalence of CST IV. Notably, women undergoing cervical maturity exhibited a higher likelihood of possessing a *Lactobacillus*-dominated profile, accompanied by lower levels of non-*Lactobacillus* microbiome profiles, when compared to individuals with unripe cervix. Furthermore, we have observed significant variations in the relative abundance of bacterial phylotypes among both the ripe and unripe groups, particularly within several low abundance genera and species. However, it remains unclear whether CST IV impedes cervical remodeling and ripening, if women with unripe cervix are more susceptible to CST IV, or whether other components within the cervicovaginal environment modulate the relationship between CST IV and cervical maturity. To further advance our understanding, future research should focus on larger prospective cohorts and investigate the mechanisms that mediate the interactions between the microbiota and the immune system during cervical remodeling and ripening. By exploring these aspects, we can gain deeper insights into the role of the microbiome in reproductive health and potential avenues for targeted interventions.

## Supporting information

S1 Fig Rarefaction curve displaying rarefaction depth by Shannon index. (TIF)

S2 Fig Comparative analysis between the groups for the Classes (A), Orders (B) Families (C) and Species (D) across all samples. Significant differences were calculated using Welch’s t-test by STAMP.
(TIF)

S3 Fig The cervicovaginal microbiome clusters by the cervix mature degree within each stratum of community state types (CST). Orthogonal Partial Least Squares Discrimination Analysis (OPLS-DA) of microbiome profiles within CST Ⅰ (A,), CST Ⅲ (B), CST Ⅳ (C).
(TIF)
