# Supplementary figures and images for: Characteristics of cervicovaginal microflora at different cervical maturity during late pregnancy: A nested case-control study
Source: PLoS One. 2024 Mar 20;19(3):e0300510. doi: 10.1371/journal.pone.0300510 (PMC10954133; doi:10.1371/journal.pone.0300510)

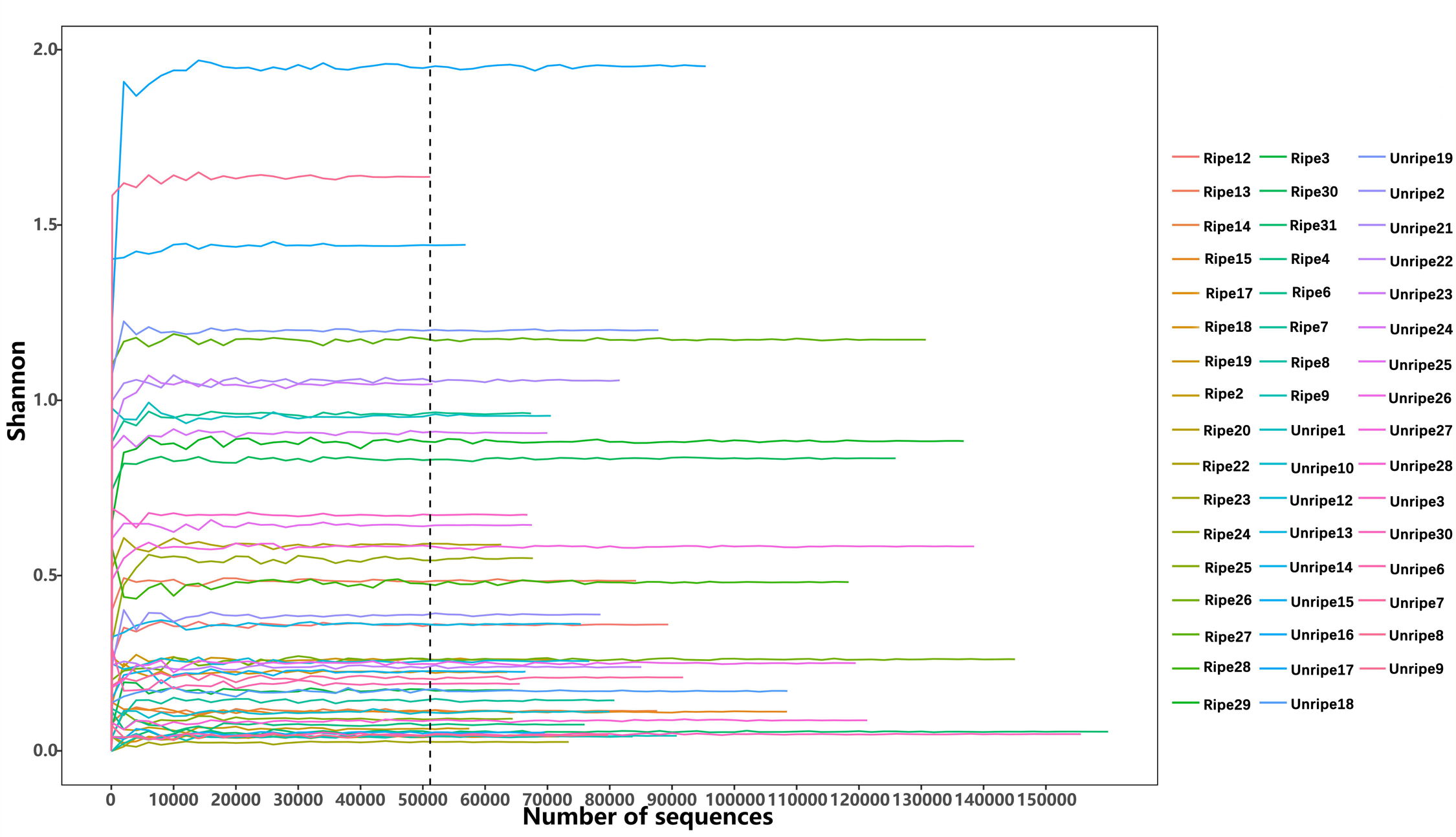

Supplement: S1 Fig — (TIF) [file pone.0300510.s001.tif]

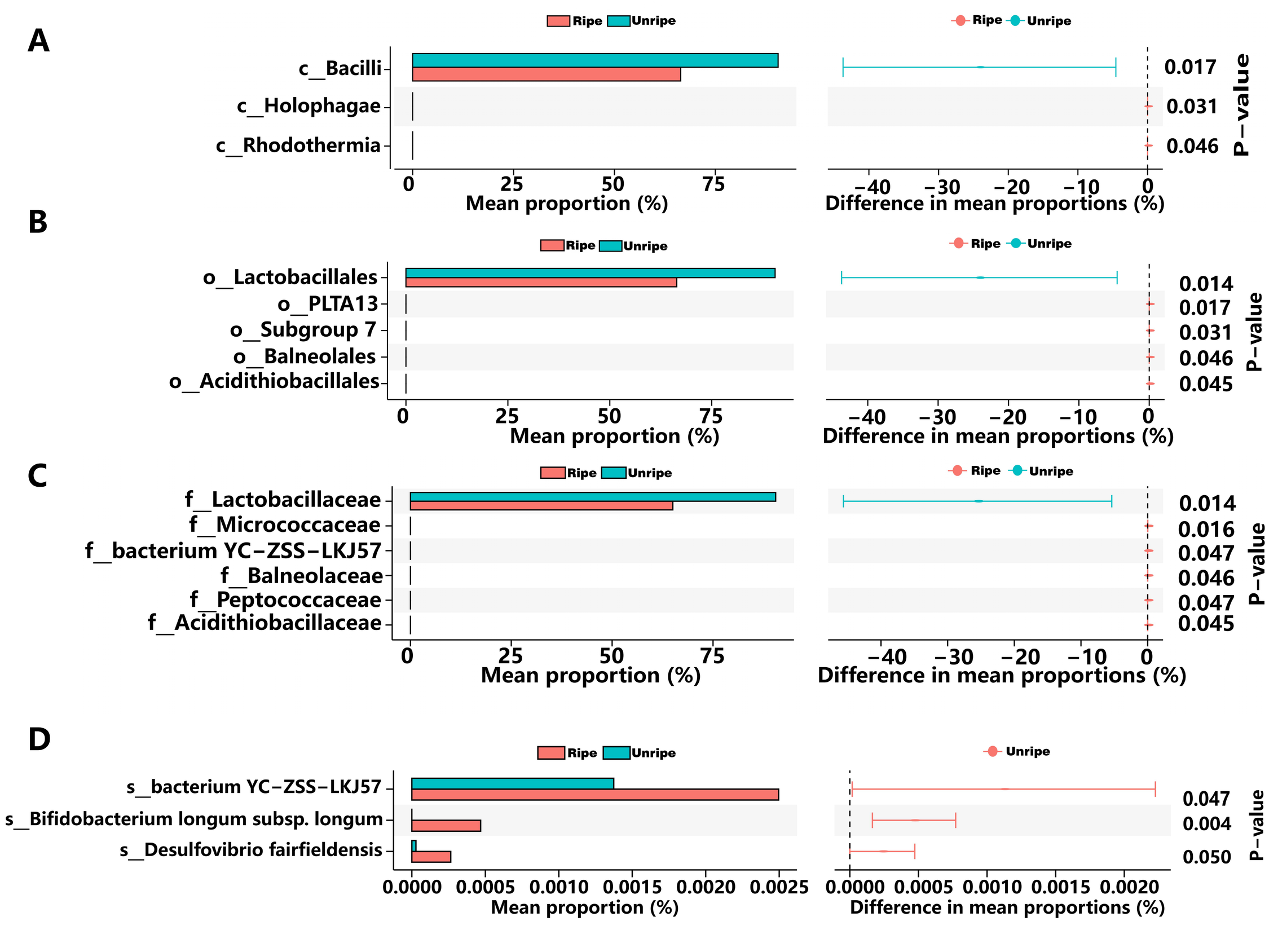

Supplement: S2 Fig — Comparative analysis between the groups for the Classes (A), Orders (B) Families (C) and Species (D) across all samples. Significant differences were calculated using Welch’s t-test by STAMP. (TIF) [file pone.0300510.s002.tif]

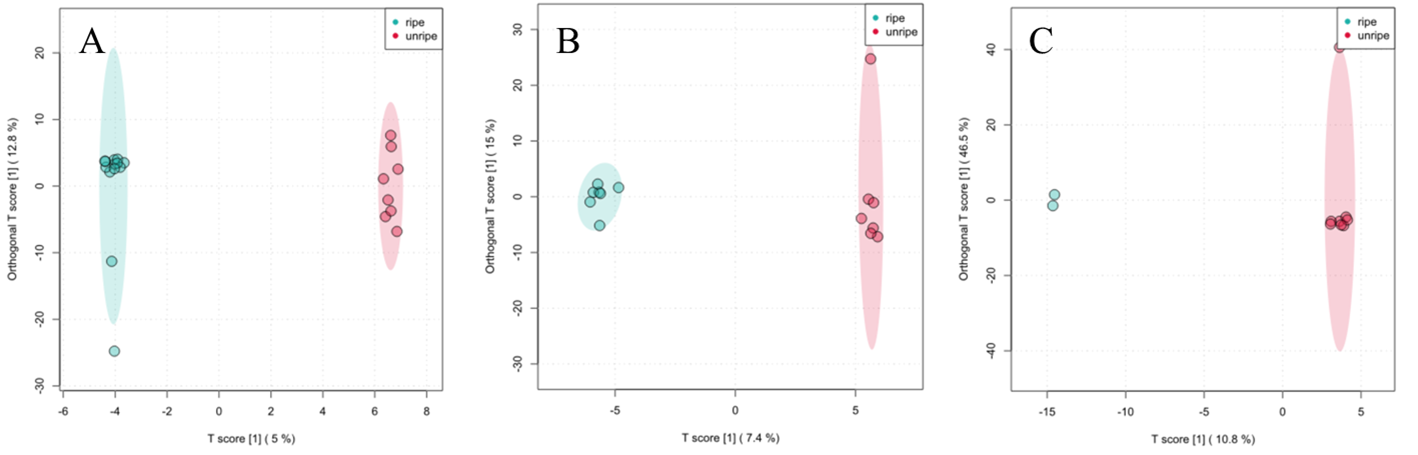

Supplement: S3 Fig — Orthogonal Partial Least Squares Discrimination Analysis (OPLS-DA) of microbiome profiles within CST Ⅰ (A,), CST Ⅲ (B), CST Ⅳ (C). (TIF) [file pone.0300510.s003.tif]
